# COVID-19’s impact on visitation behavior to US national parks from communities of color: evidence from mobile phone data

**DOI:** 10.1038/s41598-022-16330-z

**Published:** 2022-08-04

**Authors:** Charles Alba, Bing Pan, Junjun Yin, William L. Rice, Prasenjit Mitra, Michael S. Lin, Yun Liang

**Affiliations:** 1 Department of Psychology, University of Warwick, Coventry, CV5 8DR UK; 2 Eberly College of Science, The Pennsylvania State University, University Park, PA 16802 USA; 3 Department Recreation, Park, and Tourism Management, College of Health and Human Development, The Pennsylvania State University, University Park, PA 16802 USA; 4 Population Research Institute & Social Science Research Institute, The Pennsylvania State University, University Park, PA 16802 USA; 5 Department of Society and Conservation, W.A. Franke College of Forestry and Conservation, University of Montana, Missoula, MT 59812 USA; 6 College of Information Sciences and Technology, The Pennsylvania State University, University Park, PA 16802 USA; 7 School of Hotel and Tourism Management, Hong Kong Polytechnic University, Kowloon, Hong Kong, SAR; 8 L3S Research Center, Leibniz Universität Hannover, 30167 Hannover, Germany

**Keywords:** Environmental economics, Environmental impact, Socioeconomic scenarios, Sustainability, Population dynamics, Environmental social sciences, Health care, Environmental economics

## Abstract

The widespread COVID-19 pandemic fundamentally changed many people’s ways of life. With the necessity of social distancing and lock downs across the United States, evidence shows more people engage in outdoor activities. With the utilization of location-based service (LBS) data, we seek to explore how visitation patterns to national parks changed among communities of color during the COVID-19 pandemic. Our results show that visitation rates to national parks located closer than 347 km to individuals have increased amidst the pandemic, but the converse was demonstrated amongst parks located further than 347 km from individuals. More importantly, COVID-19 has adversely impacted visitation figures amongst non-white and Native American communities, with visitation volumes declining if these communities are situated further from national parks. Our results show disproportionately low-representations amongst national park visitors from these communities of color. African American communities display a particularly concerning trend whereby their visitation to national parks is substantially lower amongst communities closer to national parks.

## Introduction

The COVID-19 pandemic substantially impacted visitation patterns to U.S. national parks^1,2^. While some parks experienced record visitation^1^ as part of a national increase in outdoor recreation^3,4^, other national parks saw their visitation substantially decreased-in part due to safety concerns and travel restrictions^1,2^. The pandemic also constrained broader park visitation and outdoor recreation among some groups, including-in specific settings-urban-dwellers^5^, non-white individuals^6^, and low-income groups^6^. These constraints exacerbate previously documented constraints including limited socioeconomic resources, a lack of cultural relevance to artifacts, landscape, or recreation activities that occur in U.S. national parks, and discrimination against communities of color in these parks^7,8^. Specifically, previous research notes that the development of the National Park Service (NPS) coincided and was influenced by the rise of the U.S. eugenics movement and precipitated military-led displacement of Native Americans long-residing in many of these lands^9^. It is hypothesized that these factors contribute to the non-representative visitation in national parks among African-, Asian-, Hispanic-, and Native Americans and present a number of related and systematic issues^7–9^.

Reduced or discriminatorily constrained access to national parks among communities of color is conceptualized as a form of “environmental racism”^10^. The costs of this racially-driven discrimination are numerous. These health and environmental costs are described as follows. Floyd and Johnson^10^ advocate that we think of the distribution of health benefits from parks and outdoor recreation through an environmental justice perspective, much in the same way we might consider the distribution of health costs through hazards associated with industrial waste disposal sites or metal refineries. That is, the disproportionately low access to related health benefits among communities of color is somewhat analogous to the disproportionately frequent placement of environmental hazards near communities of color^10^. From an agency perspective, this disproportionate distribution of the benefits stemming from continued discriminatory practices in national park management also manifests in the NPS defaulting on its mission of public use for all Americans^11,12^.

Some have theorized that the pandemic may have further negatively exacerbated the relatively low level of visitation to national parks among communities of color^3^. Others have measured a widened racial disparity in urban park use during the pandemic^6^. Empirical data concerning the former has yet to be presented. The NPS collects and regularly publishes data on visitation volumes^13^. However, there is no system-wide collection or reporting mechanism for visitor demographics. Thus, estimating national park visitor demographics is usually reliant on surveys distributed to a single or a few selected park units. To understand the impact of the COVID-19 pandemic on communities of color, we elected to utilize location-based service (LBS) data obtained from mobile devices. Aggregated, anonymized location data derived from national park visitors’ mobile devices is an emerging means of understanding changes in visitation patterns^2^ and visitor demographics^14^. This study utilizes SafeGraph’s mobile phone dataset^15^. SafeGraph primarily obtains GPS locational data from millions of American mobile device holders through a variety of third-party applications^15^. SafeGraph’s inclusion of the 12-digit FIPS census block group of these mobile device holders’ community origins allows us to retrieve visitor demographics using census block group information from the American Community Survey (ACS)^16^.

Despite the perceived utility provided by mobile phone location data, minimal research on park visitation with this type of data^14,17^ has been published, in large part due to the relatively recent development of this data collection method^18^. Two exceptions are studies of park visitors in Orange County, California (U.S.)^14^ and Yellowstone National Park^17^. In both studies, the authors found demographics gathered through mobile phone location data to be relatively consistent with those reported through surveys^14^.

Thus, the aim of this paper is to identify the impacts of the COVID-19 pandemic on national park visitation among communities of color throughout mainland U.S. In doing so, we considered 932 points-of-interests (POIs) across 48 national parks in the contiguous U.S. Visitations to each respective national park were then traced to the census block origins of the visitors encompassing the entire mainland U.S. Our choice of analyzing visitation trends across the census block groups is elaborated in the Methods section below.

Through the utilization of racial demographics from visitors’ census block groups across the mainland U.S, we are able to gain insights into COVID-19’s impact on the visitation behavior of visitors from communities of color. We specifically sought to determine how COVID-19 has altered the volume of visitors to national parks and how this behavior transcends across racial communities. These racial communities include non-whites, and specifically African-, Hispanics-, Asian-, and Native American communities.

To analyze COVID-19’s impact on visitation from census block groups of color over the distance required to travel to each national park, we modeled the normalized visitations of each park using the gravity model. We will briefly explain our choice of the gravity model later in this section and elaborate on the gravity model in the methods section.

Our independent variables are the racial demographics of each census block group, COVID-19 era, and distance traveled by visitors from each census block group to each park. These racial demographics include the percent of non-whites, and specifically African-, Hispanic-, Asian-, and Native-Americans, in each census block group. Our dependent variable is the visitation count from each census block group to each national park, normalized per thousand population of the census block group. Our choice of normalizing visitation counts with the census blocks’ population is detailed in the methods section. Our variables could be illustrated as such:

1 $$\begin{aligned} \begin{aligned} \frac{visitation_{ijt}}{\left( \frac{population_i}{1000}\right) }= f(COVID~era,race_i,distance_{ij},\text {interaction terms}) \end{aligned} \end{aligned}$$

where *i* represents each census block group’s visitation counts to $j{\text {th}}$ national park during $t{\text {th}}$ month.

Using the gravity model allows us to measure the percent change in visitations from each census block group to each national park as a result of the changes from our aforementioned independent variables in Eq. (1). Specifically, in our version of the gravity model, we sought to explore how racial demographics impacted national park visitations based on the distance between the national parks and the distinct census block groups. This could be best illustrated in Eq. (2) below. Our aforementioned independent variables were selected with the goal of constructing a model that could optimally represent our goal of analyzing COVID-19’s impact on visitations from distinct racial communities^19,20^ while preserving the assumptions of the gravity model^19–22^. An elaboration of our model and the selection of variables are described in the Methods section below. In brief, the gravity model explores the influence of a socio-economic indicators on the movements of individuals over the distances between two locations. The gravity model is hence frequently used in studies involving trade^21^, tourism^19,20^, and migration^22^.

## Results

Our research seeks to model the visitation to each national park from each distinct census block group amidst the COVID-19 pandemic.

Supplementary Table S1 provides a summary of the definition of our variables while Supplementary Table S2 shows the descriptive statistics of the data used in our model. The results are best summarized in Table 1. Our results reveal that visitation figures from the broad category of non-white census block groups and the specific category of Native American census block groups have been significantly impacted due to COVID-19. This is evident from the significance (at $P < 0.05$) shown in the interaction terms involving *COVID* *era* and *race*. While our results show that COVID-19 did not impact visitation figures from African-, Hispanic, and Asian-American communities, we could still gather insights into the existing travel behavior of these communities through the other significant variables (at $P < 0.05$). Specifically, we could seek to determine how race of the census block groups and distance required to travel from each census block group to each park could impact additional changes in national park visitations.

We elaborate our findings concerning COVID-19’s impact or the overall travel behavior on visitation from each racial community in the subsequent portions of the results section.

**Table 1:** Our summary of results reveal that visitation figures from non-white and Native American census block groups have been impacted due to COVID-19. While our results show that COVID-19 did not impact visitation figures from African-, Hispanic, and Asian-American communities, we could still gather insights into the existing travel behavior of these communities through the interaction terms of other significant variables (at $P < 0.05$).

Coefficient	Race = (none)	Race = non-white	Race = African American	Race = Hispanic	Race = Asian American	Race = Native Indian
	Estimate (Robust Std Error)	Estimate (Robust Std Error)	Estimate (Robust Std Error)	Estimate (Robust Std Error)	Estimate (Robust Std Error)	Estimate (Robust Std Error)
(Intercept)	1.846*** (0.006)	1.856*** (0.006)	1.827*** (0.005)	1.852*** (0.007)	1.867*** (0.007)	1.918*** (0.016)
*COVID* *era*	0.093*** (0.013)	0.097*** (0.014)	0.092*** (0.013)	0.091*** (0.013)	0.094*** (0.015)	0.158*** (0.020)
$\ln (race)$		0.003*** (0.0003)	− 0.002*** (0.0003)	0.001*** (0.0002)	0.001*** (0.001)	0.003*** (0.0005)
$\ln (distance)$	− 0.068*** (0.001)	− 0.069*** (0.001)	− 0.065*** (0.001)	− 0.068*** (0.001)	− 0.07*** (0.001)	− 0.079*** (0.003)
$\ln (\frac{population}{1000})$	0.033*** (0.001)	0.033*** (0.001)	0.034*** (0.001)	0.033*** (0.001)	0.030*** (0.001)	0.032*** (0.001)
$COVID~era\times \ln (race)$		0.001* (0.001)	− 0.0001 (0.0003)	− 0.0003 (0.0004)	0.0001 (0.0003)	0.003*** (0.0004)
$COVID~era\times \ln (distance)$	− 0.016*** (0.002)	− 0.017*** (0.002)	− 0.015*** (0.002)	− 0.015*** (0.002)	− 0.016*** (0.002)	− 0.026*** (0.003)
$\ln (race)\times \ln (distance)$		− 0.0005*** (0.0001)	0.0002*** (0.00004)	− 0.0001** (0.00004)	− 0.016*** (0.002)	− 0.00045*** (0.0001)
$COVID~era\times \ln (race)\times \ln (distance)$		− 0.0003* (0.0001)	0.0001 (0.0001)	0.0001 (0.0001)	− 0.00001 (0.00004)	− 0.0004*** (0.0001)
N = 221526						
Psuedo Adj R-squared (within)	0.217	0.217	0.223	0.229	0.229	0.217
Psuedo Adj R-squared (between)	0.893	0.948	0.97	0.983	0.984	0.987

Signif. codes: 0 ‘***’ 0.001 ‘**’ 0.01 ‘*’ 0.05.

**Figure 1:**
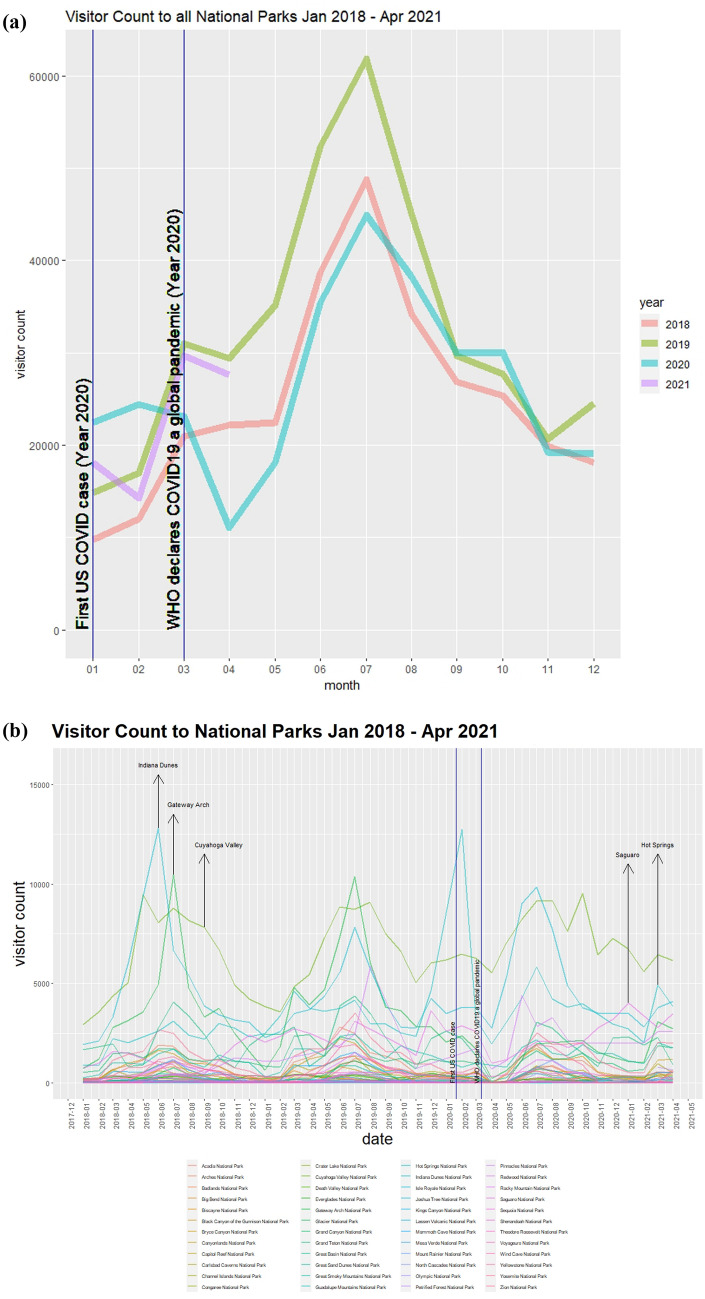
Overall visitation trends from Jan 2018 to Apr 2021. (**a**) On the top compares the changes in visitations from each year from 2018 to 2021 in visitations of all national parks. (**b**) On the bottom compares the changes in visitations for each national park.

Figure 1a illustrates the overall visitation to all national parks. The purple line of the figure shows that total national park visitation took an initial slump after COVID-19 cases surged in the U.S. However, it was followed by sharp increases in the subsequent months, starting in April 2020. The month of October 2020 recorded higher visitation figures compared to the same period of the preceding years of 2018 and 2019. This signalled the stabilization of national park visitation and support the “revenge travel” phenomenon. The “revenge travel” phenomenon is the idea that individuals are eager to explore natural spaces upon being confined or restricted to their personal surroundings imposed by COVID-19 restrictions^23,24^.

The visitation to each respective national park and its effects amidst the COVID-19 pandemic is illustrated in Fig. 1b. Supplementary Fig. S1 shows that while most national parks witnessed an overall decrease in visitor volume following the COVID-19 crisis, a few parks witnessed an increased visitation. For instance, Cuyahoga Valley, Indiana Dunes, Redwood, and Yellowstone all witnessed increased annual visitation relative to the preceding year.

### How has COVID-19 impacted visitation behavior to national parks from racially diverse communities

#### Overall visitation to national parks

Due to a substantial increase to a few large national parks (Cuyahoga Valley, Indiana Dune, Redwood, and Yellowstone), the first three of which are within close distance to population centers, overall national park visitation significantly increased (at $P < 0.05$) amidst the first year of the COVID-19 pandemic, assuming the distance travelled remains constant. This is evident across the results displayed in Table 1. However, most national parks witnessed a decrease. To be more precise, on average, assuming the distance travelled remains constant, each individual national park is expected to witness an average increase of 0.0971% in visitation from each census block group following the COVID-19 pandemic.

**Figure 2:**
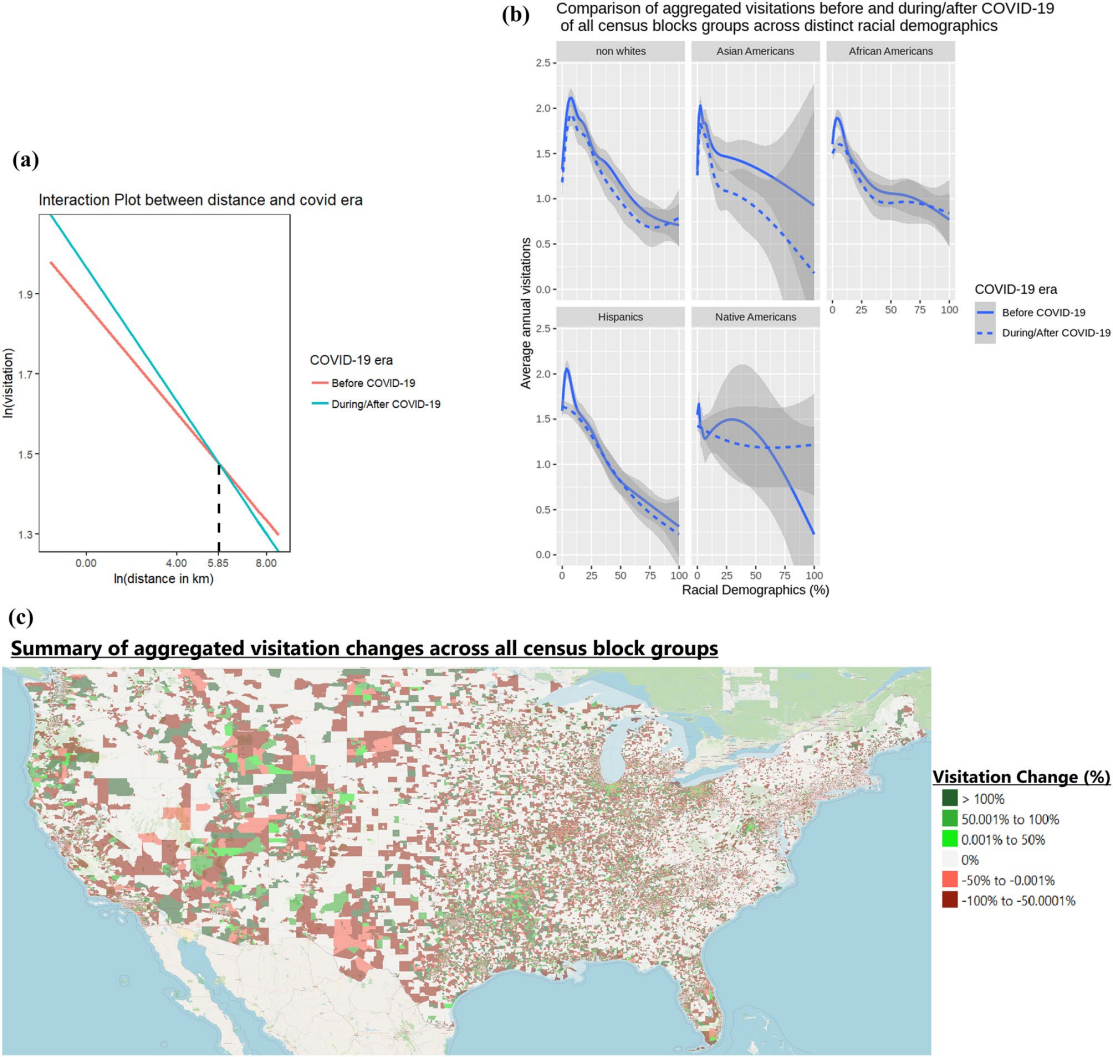
Visualizations of the overall impact of COVID-19 towards park visitations. (**a**) on the left reflects the interaction plot between distance and COVID-era. (**b**) On the top-right, reflects the impact of annually aggregated visitations across distinct racial demographics due to COVID-19. (**c**) On the bottom-right, maps the overall impact of COVID-19 on visitations from all census block groups across contingent USA. The map was generated using QGIS^25^, with the map layers being facilitated by OpenStreetMaps^26^.

From the perspective of visitations from the census origins of visitors, the impact of COVID-19 on visitations across distinct racial groups could be best illustrated in Fig. 2b. Similarly, the map in Fig. 2c allows us to geographically illustrate COVID-19’s overall impact on visitations across census block groups spanning mainland America.

Taking distance into account, our results show that visitation rates have decreased amongst visitors requiring longer travel distances following the COVID-19 pandemic. The opposite is true for visitors requiring shorter travel distances. We provide specific details concerning the travel distances in the next paragraph. These are best illustrated in the interaction plot of the fitted model contained in Fig. 2a. This fitted model’s interaction plot allows us to visualize COVID-19’s effect on park’s visitation based on the distance travelled by the visitors to each park. The raw estimates displaying the relationship between COVID-19 and distance could be illustrated in Supplementary Fig. S2. Due to the massive number of raw points, as evident from Supplementary Fig. S2, we have elected not to overlay them in the interaction plots throughout this article.

Specifically, each park would witness a $\left[ 0.093-0.016*\ln (distance_{ij})\right]$% change in visitations from a census block group (*i*) due to COVID-19. An explanation on the derivation of this formula could be referenced in supplementary section S1. For instance, if a $j{\mathrm{th}}$ park is situated within 1 kilo-meters from $i{\mathrm{th}}$ census block group, the park would witness an average of 0.093% ($0.093-0.016*\ln (1)$) increase in visitors from census block group *i* as a result of the pandemic. However, beyond 347 km (or $e^{5.85}$), as evident from Fig. 2, the park would begin to witness decreases in visitation rates from census block group (*i*) when compared to that of the pre-COVID era. The 95% confidence interval for this distance threshold, based on the Delta Method^27^, falls between 333 km and 372 km. This confidence interval could be relied upon to reflect the heterogeneous nature of national parks visitations^28^. Distance decay rates are the amount of decrease in visitations due to a larger distance. This result shows an increased distance decay rate amid the pandemic. The distance of 347 km (equivalent of 216 miles) takes around 3-4 hours’ drive time. This seems to be the demarcation between a day-trip to visit a national park versus an overnight trip.

#### COVID-19 impact on visitation behavior from non-white communities

**Figure 3:**
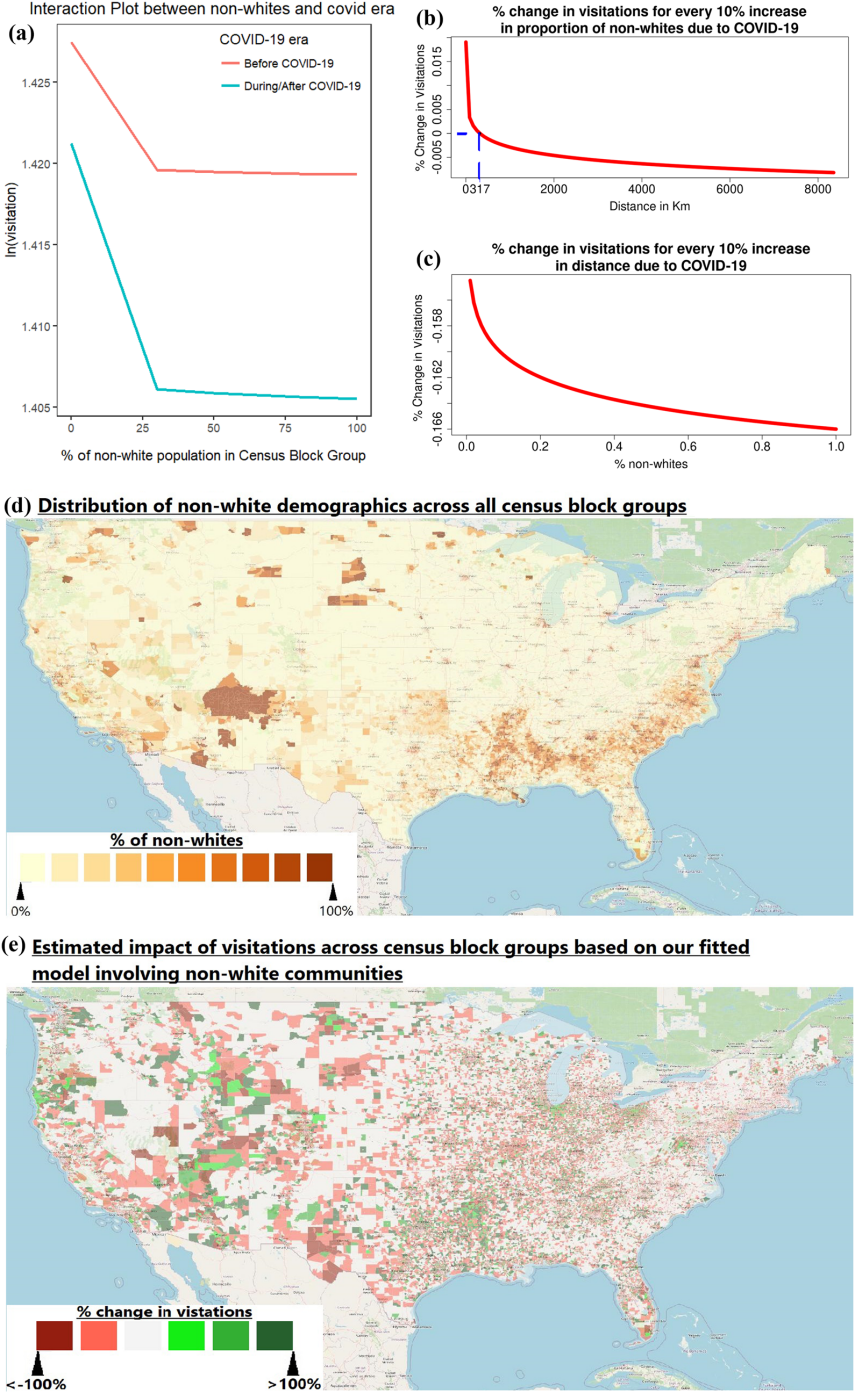
Illustration of the of impact of COVID-19 towards visitations to national parks from non-white communities. (**a**) On the left reflects the interaction plot of percent of non-whites in a census block group and its impact on park visitations due to COVID-19. (**b**,**c**) On the right reflects changes of visitation to each national park due to COVID-19 for every 10% increase of the proportion of non-whites and distance travelled respectively. (**d**,**e**) Allows us to compare the non-white demographics across all census block groups and our estimated impact on their overall visitation figures to national parks due to COVID-19, respectively. The maps were generated using QGIS^25^, with the map layers being facilitated by OpenStreetMaps^26^.

We have strong evidence to suggest that COVID-19 has caused communities with more non-white populations to experience a more severe decrease in visitation to national parks. This relationship is significant (at $P < 0.05$), as illustrated in the interaction plot in Fig. 3a. This interaction plot allows us to visualize COVID-19’s impact on park visitation from each census block group based on the proportion non-white’s in a census block group.

Figure 3b shows that COVID-19’s impact on park visitation amongst populations with higher proportion of non-white residents varies according to distance required to travel from the census block groups. Each national park would witness a $\left[ 0.0014-0.00025*\ln (distance_{ij})\right] \%$ change for every percent change in the proportion of non-white residents from census block *i* due to the COVID-19 pandemic. Explanation on the derivation of this formula can be referenced in supplementary section S2. This impact depends on the distance between a community and a national park: within 317 km, there are actually increases in visitation during the pandemic from a more diverse community; beyond that distance, visitation rates drop with a more diverse population. The 95% confidence interval for this distance threshold falls between 315 km to 321 km.

To interpret the results in the context of community composition, in Fig. 3c, the values in the y-axis represent distance decay rates. As a result of the pandemic, a particular park would observe $\left[ -0.016-0.00025*\ln (non\_white_i)\right]$% change in visitation rate for every additional percent change in distance travelled from census block *i* to national park *j*. Explanations on the derivation of this formula are detailed in supplementary section S2. The figure shows that more diverse communities are less likely to travel further as a result of the COVID-19 pandemic, showing a higher distance decay rate.

Figure 3d,e allow us to geographically illustrate the non-white communities which were predicted to witness the largest impacts of visitations to national parks as a result of the COVID-19 pandemic.

#### COVID-19 impact on visitation behavior in African American communities

We have no evidence to suggest that COVID-19 has impacted the visitation behavior from communities with a higher percentage of African Americans.

**Figure 4:**
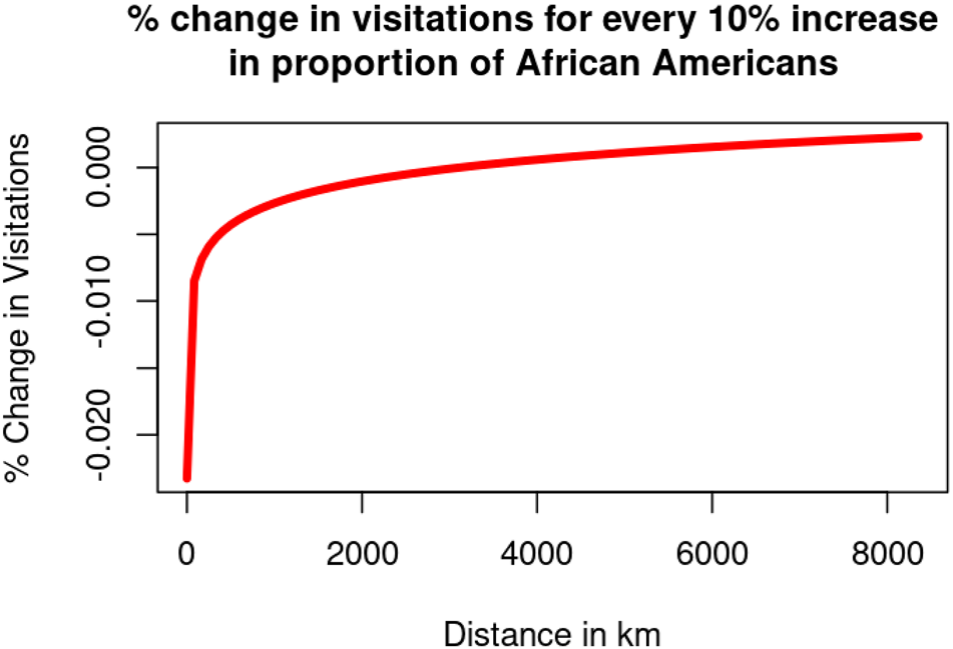
Changes visitation to each national park for every 10% increase of the proportion of African Americans.

Our results reveal that regardless of the COVID-19 pandemic, national parks overall witness lower visitation counts from communities with a larger percentage of African Americans. To interpret our results in the context of distance, in Fig. 4, the values in the y-axis roughly represent the impact of African American majority communities. A park would witness a $\left[ -0.0018+0.00024*\ln (distance_{ij})\right]$% change in visitation from census block *i* for every percent increase of the proportion of African-American population. An explanation on the derivation of this formula could be referenced in supplementary section S3.

However, as evident from Fig. 4, regardless of COVID-19, the rate of decrease in visitors from communities with a larger percentage of African Americans improves as the parks are located further away. This potentially suggests that African Americans are more likely to maintain visitation level to national parks further away than to those parks in close proximity.

#### COVID-19 impact on visitation behavior from Hispanic communities

We have no evidence to suggest that COVID-19 has impacted the visitation behavior amongst visitors from Hispanic communities.

Despite yielding a positive coefficient for “$\beta _2*\ln (Hispanic_i)$”, we have to factor in the significance amongst the interaction terms involving Race and Distance. We found that regardless of COVID-19, a park would witness a $\left[ 0.00066-0.00012*\ln (distance_{ij})\right]$% change in visitation from census block *i* for every percent increase in Hispanic population in that block. An explanation on the derivation of this formula can be referenced in supplementary section S3.

**Figure 5:**
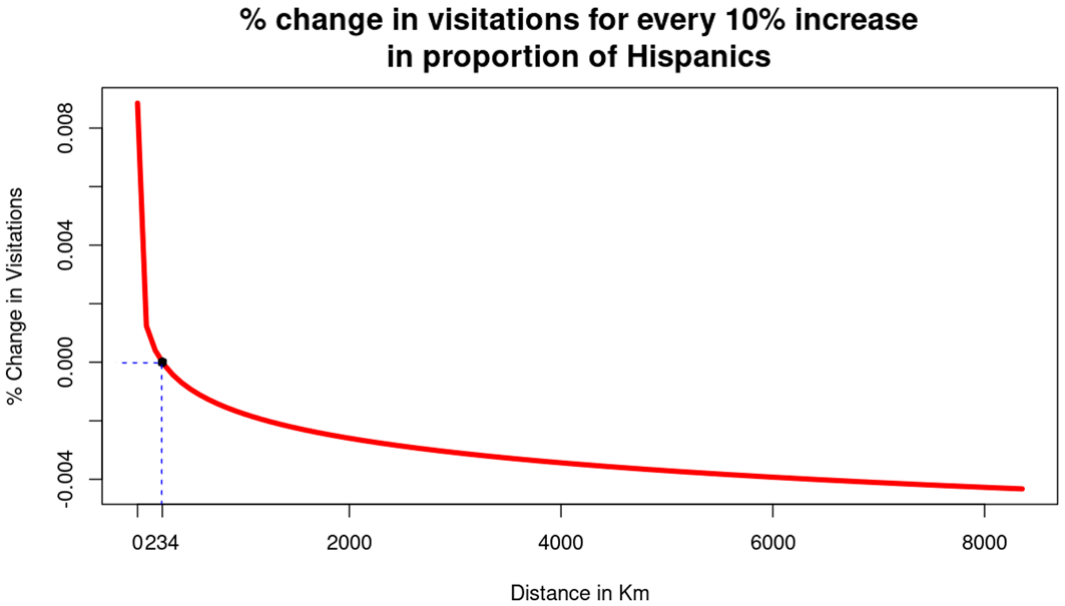
Changes visitation to each national for every 10% increase of the proportion of Hispanics.

As per Fig. 5, regardless of COVID-19, national parks would witness higher visitation from a community with a higher percentage of Hispanic population, if they are located less than 234 km away, otherwise, visitation from those communities would decrease with an increased Hispanic population. This suggests that Hispanic communities are likely to visit national parks nearby but are less likely to travel longer distances (i.e. $>234$ km) to visit national parks. This indicates that the disparity of national park utilization for Hispanic visitors largely due to access to national parks farther away.

#### COVID-19 impact on visitation behavior from Asian American communities

We have no evidence to suggest that COVID-19 has impacted the visitation behavior amongst visitors from Asian American communities.

**Figure 6:**
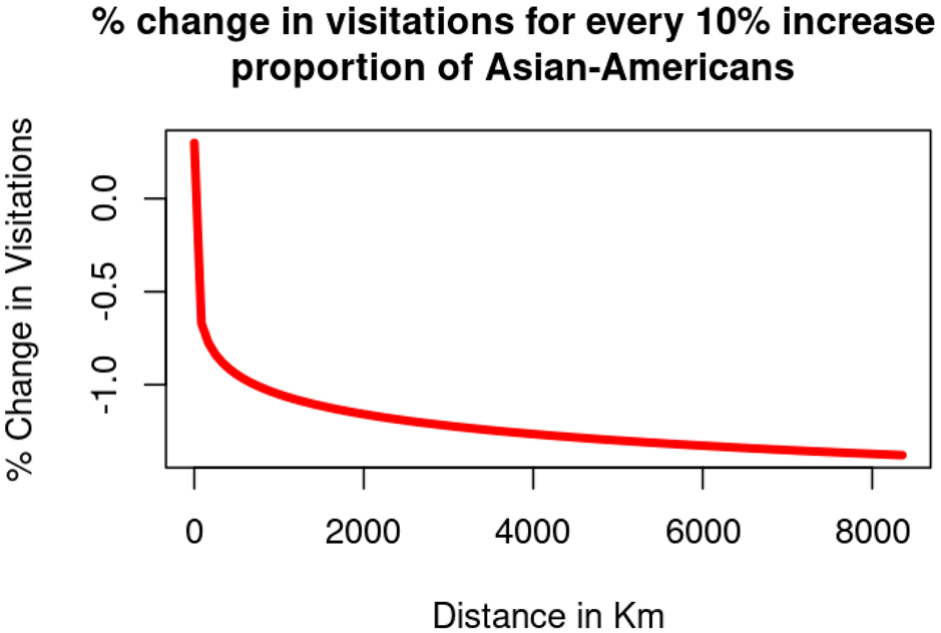
Changes in visitation to each national park for every 10% increase of the proportion of Asian Americans.

Despite yielding a positive coefficient for “$\beta _2*\ln (Asian\_American_i)$”, we have to factor in the significance measures of the interaction terms involving Race and Distance. Thus, to interpret our results in the context of distance, regardless of COVID-19, a national park would witness a $\left[ 0.0012-0.016*\ln (distance_{ij})\right]$% change in visitation from census block *i* for every percent increase of the proportion of Asian-Americans. As best illustrated in Fig. 6, unless a national park is situated extremely close (i.e. $<1.08$ km) to a census block group, the census block group would witness more reduced visitation for every percent increase in its Asian American population. An explanation on the derivation of this formula can be referenced in supplementary section S3.

This suggests that Asian American are generally unincentivized from visiting national parks but they are more likely to visit national parks in their community (within 1 km). Thus, the disparity for accessing national parks for Asian Americans are across all distances except the ones extremely close-by.

#### COVID-19 Impact on Visitation Behavior from Native American communities

**Figure 7:**
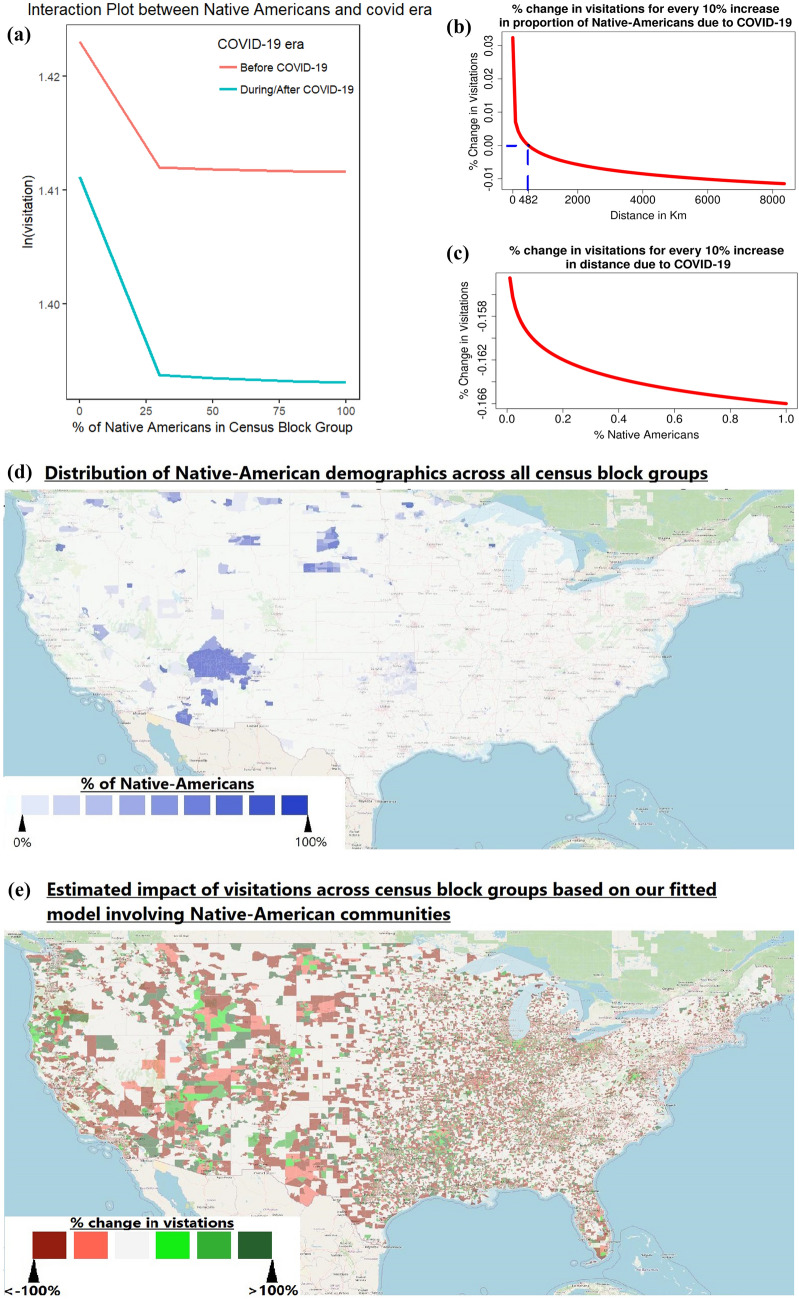
Illustration of the of impact of COVID-19 towards visitations to national parks from Native American communities. (**a**) On the left reflects the interaction plot of percent of Native Americans in a census block group and its impact on park visitations to national parks due to COVID-19. (**b**,**c**) On the right reflects changes of visitation to each national park due to COVID-19 for every 10% increase of the proportion of Native Americans and distance travelled respectively. (**d**,**e**) Allow us to compare the demographics of Native-Americans across census block groups and our estimated impact on their overall visitation figures to national parks due to COVID-19, respectively. The maps were generated using QGIS^25^, with the map layers being facilitated by OpenStreetMaps^26^.

We have strong evidence to suggest that visitation rates from Native American communities has decreased due to COVID-19. This relationship is significant (at $P < 0.05$). This can be best illustrated in the interaction plot in Fig. 7a. This interaction plot allows us to visualize COVID-19’s impact on park visitations from each census block group based on the proportion of Native Americans in the census block groups.

To interpret the results in the context of distance, each national park would witness a $\left[ 0.0025-0.0004*\ln (distance_{ij})\right] \%$ change from a community for every percent change in its Native Indian population from census block *i* due to the COVID-19 pandemic. These changes are best illustrated in Fig. 7c. An explanation on the derivation of this formula can be referenced in supplementary section S2. The value shows that distance plays a role in this impact: an increase in visitation happens for a community with a larger percentage of Native Americans if the national park is within 482 km; it is a decrease otherwise. The 95% confidence interval for this distance threshold, based on the Delta Method^27^, falls between 480 km and 485 km.

To interpret the results in the context of community composition, as a result of the pandemic, a particular park would observe $\left[ -0.026-0.0004*\ln (Native\_American_i)\right] \%$ change in visitation rate for every additional percent change in distance travelled from census block *i* to national park *j*. These changes are best illustrated in Fig. 7b. An explanation on the derivation of this formula are similar to that of supplementary section S2. This shows a higher distance decay effect for a community with a larger Native American population.

Figure 7d,e allow us to geographically illustrate the Native-American communities which were predicted to witness the largest impacts of visitations to national parks as a result of the COVID-19 pandemic.

## Discussion

While the aforementioned results reflect an overall increase in national park visitation during the first year of the pandemic, compared to the previous year, this is mainly due to a few large national parks in close proximity to population centers. In addition, the sharp increase in visitation occurring in April and May 2021 suggests that many seized the opportunity to visit in the spring and summer of 2021-as parks began opening up from initial pandemic-related closures^1^. This increase in visitations in the latter periods of Spring and Summer 2021 was driven in part by an increased willingness to travel^29^. This is due to the reduction of the perceived threat of COVID-19 as the number of vaccinated individuals increased^29^. However, the fact that these increased levels of visitation only occurred amongst census block groups situated less than 347 km from the respective national park suggests that many residents visited the national parks within their respective states or regions-rather than pursuing long-distance or cross-country visits to national parks. As a point of reference, the width and length of the geographic extent of New York State are 530 km and 455 km, respectively, whereas the width and length of California State are 1,240 km and 400 km^30^.

This finding is not surprising given the limited air travel due to safety concerns likely discouraged individuals from taking long-distance trips^31^. In addition, the distance could also be the range of a day trip instead of an overnight trip; the latter is usually perceived with a higher risk of exposure to COVID-19. Furthermore, the distinct state-wide travel restrictions would certainly defer or hinder people from pursuing cross-country, or—in some cases—even regional travels to visit national parks. These findings affirm the instrumental impact of geographical variations surrounding state-level COVID-19 policies towards the observed changes concerning park visitations. For instance, while encountering different levels of pandemic development, states in the U.S. implemented lockdown, social distancing, and masking policies at different times^32^. These state-level policy dynamics inevitability created complexity and confused travelers. Websites have been setup to inform travelers about this dynamic. Hence, it was natural for travelers to forego cross-state travel for trips to parks within their own states.

Importantly, among specific communities of color, only communities with a high percentage of Native Americans saw a significant impact (at $P < 0.05$) on national park visitation during the COVID-19 pandemic. However, an omnibus examination of communities with a larger non-white population found that the COVID-19 pandemic significantly impacted (at $P < 0.05$) overall minority visitation to national parks, whereas visitation to a national park from a more diverse community decreased more as the portion of non-white residents within that census block group increased.

The results from non-white communities show a concerning trend: a visitation from these communities was already disproportionately underrepresented prior to COVID-19, and this under-representation has only worsened following the COVID-19 pandemic, depending on the distances to national parks. Our results align with previous research from a study of park visitation in New York City^6^ showing similar results and follow the predictions of Xiao et al.^3^.

From an environmental justice perspective, this means that the COVID-19 pandemic has led a widening gap in the already lopsided portion of benefits derived from national parks going to white communities^10^. This widening gap could have been driven by the racial contrasts surrounding risk perceptions of COVID-19 and its impact on an individual’s willingness to travel. Because ethnic minorities perceived COVID-19 as a greater threat compared to their white counterparts^33^, this may have induced inertia in their reluctance to travel^34^. These findings also reflect the existing spatial inaccessibility of parks from minority populations^8^—because parks are disproportionately situated further away from non-white communities^8^, visitations from non-white communities were more susceptible to decline amidst the COVID-19 pandemic. This inequitable impact could be visually illustrated in the maps of Fig. 3d,e. With a few exceptions, most major national parks are located in relatively remote places. Thus, it takes economic resources and dispensable time to engage in this types of travel where many minority groups were lacking^35^.

Additionally, the disproportionate impact on visitations from non-white communities is not be surprising given that numerous studies that suggest COVID-19’s inordinate economic impact on non-white and racially diverse communities^36,37^. Thus, the alleviation of impacted visitor representation from non-white communities due to COVID-19 revolves around mitigating economic devastation caused by the pandemic towards these communities^36,37^.

Similarly, COVID-19 has devastated visitation figures amongst visitors from Native American Communities. This can be attributed to the fact that these communities were already disproportionately affected by the COVID-19 pandemic due to a relatively high frequency of underlying chronic health conditions among Native Americans, a deficient in institutional resilience, poor access to quality healthcare, and a lack of social trust^38,39^. Furthermore, as some Native American communities in the western U.S. are reliant on the tourism economy, the COVID-19 pandemic considerably exacerbated existing economic hardships^40^. It is thus not surprising that the pandemic caused a considerable decrease in national park visitation from Native American communities farther away.

Communities with a large Native American population actually are more likely to visit nearby national parks, but not national parks more than 482 km away. Bridging the disproportionate impact of park visitation from farther communities with a large Native American population due to COVID-19 centers around mitigating the inordinate economic devastation of COVID-19. Thus, existing literature supporting increased financial assistance towards these communities^39,41^ could aid the circumvention of the impacted visitation from Native American communities.

While visitation demographics from African-American communities remain unchanged as a result of the COVID-19 pandemic, our finding of disproportionate visitation patterns amongst African American communities are of utmost concern towards the diversity of park visitors. Further, as evident from Fig. 4, we witnessed a distinct visitation trend amongst African American communities: the closer the park is situated to a community, the lower the likelihood of visitation. This is a somewhat unintuitive finding but leads us to conclude that African American communities are more likely to engage in long-distance, cross-country travel to visit national parks, rather than visiting more proximate national parks. This finding follows previous findings denoting that African Americans are more likely to visit national parks that are relevant and celebrate the history of their community^8^. Therefore, they may be willing to travel further to visit these relatively few NPS units as opposed to visiting proximate NPS units that may not be culturally relevant^7^.

Our intuitive findings towards visitation patterns amongst African American communities could be translated to policy practices to welcome more African Americans to visit national parks. National parks could increase efforts to celebrate the histories of African American communities by organizing more inclusive events, as has been effectively demonstrated in other leisure-related destinations^42,43^.

While visitation trends from Asian American communities are more conventional (i.e., the more distant the national park, the more dramatic the decrease in representation) compared to African American communities, the fact that they experienced dramatically reduced likelihood of visitation unless the national park situated practically next to their community (i.e., $< 1.08$ km) is an additional cause of concern towards the attainment of racial diversity of national park visitation. Hispanic American communities exhibit a similar trend to Asian American communities, albeit less dramatic. Unless the national park is relatively close (i.e., $< 234$ km) to a census block group, the census block group would witness reduced visitation for every percent increase of the proportion of Hispanic Americans.

Likewise, our findings toward Asian and Hispanic American communities suggest the preference of day trips or short inter-state travels of close proximity to these communities. While a myriad of factors is certainly influential toward this visitation behavior, existing literature extracting disparities amongst the benefit of paid leave amongst Asian^44^ and Hispanic American^45^ employees could potentially explain this behavior. Thus, the attainment of equity in paid leave benefits could potentially entice Asian and Hispanic Americans to spend more time on leisure activities situated within further proximity. Furthermore, the combination of restricted mobility within Hispanic communities^46^ and the lack of knowledge regarding national parks—a previous study found that only 32% of Hispanics could name a U.S. national park^7^—could possibly explain the visitation behavior amongst Hispanics.

## Methods

### Materials

#### Data sources

Supplementary Table S1 summarizes the definitions of all the variables and Supplementary Table S2 displays the descriptive statistics of the variables. A detailed description of our data sources is summarized in Supplementary Table S3.

In summary, our mobile phone data, containing Jan 2018 to Apr 2021 visitation records to each national park and the visitors’ respective census block groups, are courtesy of SafeGraph Inc^47^. The geographical boundaries of national parks that are used to extract records only relevant to national parks are provided by the NPS Land Resources Division^48^. Finally, the racial and population demographics of each census block group are provided by the 2015-2019 American Community Survey (ACS)^16^.

The utilization of each distinct dataset towards the extraction of our materials of interest are elaborated in the subsequent sections.

#### Validation of SafeGraph’s mobile-phone dataset

The validation of SafeGraph’s mobile-phone dataset in its application to national parks has been previously validated by Yun et al^17^. Specifically, Yun et al’s^17^ work showed a close resemblance between the NPS visitor use survey and SafeGraph’s mobile-phone dataset in terms of visitation counts, temporal visitation patterns, racial demographics, and state-level residential origins of the visitors to Yellowstone National Park. However, SafeGraph’s POI classification of “National Parks” remains inconsistent with the NPS’s official definition of National Park. To circumvent this problem, we have utilized shapefiles courtesy of the NPS OpenData^48^ to extract the most visited POIs that fall within the shapefiles of each respective “National Park”. This process would be detailed in the subsequent sub-sections below.

#### Selection of mainland US national parks

We adopted the official and formal definition of national parks as defined and listed by the NPS System^49^.

We selected national parks within the 48 states encompassing the contiguous U.S. We chose to omit the parks that fall within the states of Alaska, Hawaii, Puerto Rico and other US minor Islands considering the fact that air travel is a necessity for out-of-state visitors to visit these select parks. These separate travel behavioral patterns could result in confounding variables towards our analysis, particularly since air travel faced major disruptions amidst the COVID-19 pandemic^50^.

It is worth noting that New River George National Park was declared as a national park only following the COVID-19 pandemic^51^. Hence, it is excluded from our study.

Finally, we lack the data availability for White Sands National Park and Dry Tortugas National Park. The former is due to its proximity to White Sands Missile Range and security concerns on mobile device data^52^. The latter’s lack of data availability could be attributed to the fact that the park is an island off the coast of Key West, FL^53^.

Henceforth, we included a grand total of 48 national parks in our study.

#### Extraction of POIs

We selected our points-of-interests (POIs) based on the dataset made available by SafeGraph^47^. While SafeGraph does provide its own classification of “national parks”, its classification methodology remains inconsistent with the NPS’s official definition and formal list of “national parks”^17,49^.

Hence, we extracted POIs that fall within the encompassed polygon shapefiles of each respective national park. The polygon shapefiles are courtesy of the NPS OpenData^48^.

We then selected the POI with the highest average monthly visitation records for each distinct national park.

The choice to select the POI with the highest visitation record could be attributed to the fact that a brief analysis reveals that in many parks, the top 5 most populated POIs tends to fall within the same vicinity^17^. Specifically, the top 5 most populated POIs for many large national parks, like Cuyahoga National Park, Indiana Dunes National park, and Yellowstone National Park, typically encompass the areas surrounding the park entrances^17^. This remains rational since visitors would have to pass through park entrances to enter the parks and gain access other areas of the park. Hence, selecting only the POI with the highest visitation record for each park prevents us from making duplicate counts from separate POIs.

#### Computing census block group-based racial demographics

The aforementioned Safegraph^47^ data provides us with the census block group origins of the visitors to each distinct POI. The census block group origins are identified by its 12-digit Federal Information Processing Standard (FIPS) code. We are thus able to retrieve our racial demographics of interests (% of non-whites, % of African-, % Hispanics-, % of Asian-, and % Native Americans) pertaining to each visitors census block origins.

Our study only considered all visitations across mainland U.S. As such, we have excluded visitors from Hawaii, Alaska, Puerto Rico and other minor US islands for their visitation patterns are expected to be abruptly disrupted following the pandemic due to restrictions put in place from air travel^50^. This decision would prevent the effects of confounding variables and avoid drastically skewing our data.

#### Computing distance travelled by visitor to each national park

Likewise, we obtain the variables of distance through the utilization of the Haversine formula^54^ between the POIs coordinates and the centroids of the visitors census block group. We standardize the units of distance to kilometers in our analysis.

#### Categorization of visitation records falling before and after COVID-19

We categorize pre-COVID era as any time-period that occurs prior to the month of March 2020. Hence, we classify the COVID era as any time period from the month of March 2020 onward. We selected March 2020 for it was the month in which the UN declared COVID-19 a global pandemic^55^. This declaration was proceeded by numerous state and local lockdown measures which drastically impacted American commerce^56^ and the lifestyles of many Americans^57^.

### Methods and Model

#### Offsetting visitation counts with the census block group population

We offset our dependent variable of visitation counts per census block population because racial demographics of the visitors’ census origins are measured at a census block level. This allows us to account for the fact that one would naturally expect higher visitation counts from more populated census block groups. Hence, the visitation counts per thousand population of the census block group would serve as a function of our independent variables (COVID-19 era, distance and racial demographics). This could be illustrated in Eq. (1) in the introduction section.

#### Gravity Model

We incorporated gravity models into our methodology. In the context of tourism, the gravity model explores the behavior and travel patterns over distances between two unique POIs.

The gravity model was adopted from Newton’s law of universal gravitation in physics^58^. Newton’s law of universal gravitation states that distance and mass determine the gravitational forces between two objects. The gravity model has since been adapted by numerous disciplines in the social sciences. These topics include trade^21^, tourism^19,20^, and migration^22^. For instance, the gravity model is popular in studies involving bilateral trade^21^. This is because the gravity model allows economists to measure how specific economic indicators (such as GDP) could attract trade between two countries, given the distances between them^21^.

We thus elected to use the gravity model because it best represents our research theme of seeking to analyze the changes in visitations to national parks amongst individual racial communities across the U.S. Henceforth, the gravity model allows us to best analyze the change in visitations from different racial communities to each specified national park given the required distance of travel. The selection of our variables, in seeking to optimally represent the gravity model, while preserving its assumptions, would be elaborated in the subsequent subsections below.

Our application of the gravity model works as such: given $i{\mathrm{th}}$ census block group and $j{\mathrm{th}}$ national park where $\alpha _k$ symbolizes each respective coefficient towards the determined independent variable, the gravity model could be demonstrated as such:

2 $$\begin{aligned} \frac{visitation_{ijt}}{\left( \frac{population_i}{1000}\right) }\propto \frac{race_i^{\alpha _1}*interaction\_terms^{\alpha _2}}{distance_{ij}^{\alpha _3}} \end{aligned}$$

which can be remodelled as:

3 $$\begin{aligned} visitation_{ijt}\propto \frac{race_i^{\alpha _1}*(interaction~terms)^{\alpha _2}*\left( \frac{population_i}{1000}\right) ^{\alpha _4}}{distance_{ij}^{\alpha _3}} \end{aligned}$$

using natural logarithms could be transformed to:

4 $$\begin{aligned} \ln (visitation_{ijt})\propto {\alpha _1}\ln (race_i)+{\alpha _2}\ln (interaction~terms)+\alpha _3\ln (distance_{ij})+ {\alpha _4}\ln \left( \frac{population_i}{1000}\right) \end{aligned}$$

#### Model Specification

The gravity model is incorporated using panel data with interaction terms^19,21^. Incorporating panel data allows us to control for unobservable individual effects^19,21^, such as time invariant monthly and seasonal fluctuations in park visitations, as best illustrated in the peaks and troughs witnessed in Fig. 1. The interaction terms allows us to measure the impact of COVID-19 towards our selected predictors. Specifically, the random-effects panel approach was selected in favor of the fixed-effects panel model and the pooled ordinary least squares (OLS) model as evident by the results of the F-tests, Hausman’s Chi-Squared, and the Breusch-Pagan (BP) Lagrange Multiplier^59^ tests displayed in Supplementary Table S4.

This results in Eq. (5), given each $i{\mathrm{th}}$ census block group’s visitation to $j{\mathrm{th}}$ national parks during $t{\mathrm{th}}$ month over specified race $race_i$.

5 $$\begin{aligned} \begin{aligned} \ln \left( visitation_{ijt}\right)&= \beta _0+\beta _1(COVID~era)+\beta _2[\ln (race_{i})] +\beta _3[\ln (distance_{ij})] +\beta _4\left[ \ln \left( \frac{population_{i}}{1000}\right) \right] \\ {}&\quad +\,\beta _5[COVID~era\times \ln (race_{i})] +\beta _6[(COVID~era\times \ln (distance_{ij})] +\beta _7[\ln (distance_{ij})\times \ln (race_i)] \\ {}&\quad +\,\beta _8[(COVID~era\times \ln (distance_{ij})\times \ln (race_i)]+V_{ijt} \\ \end{aligned} \end{aligned}$$

The assumptions of log-linearity and multi-collinearity^19–21^ in our specified model, per Eq. (5), have been tested and could be referenced in Supplementary Table S5.

#### Consideration of variables in our model

We explored using the size area (in km$^2$) of each respective park, instead of distance travelled, as the denominator of our gravity model per Eq. (2). However, the substantially lower $R^2$ values obtained when using a park’s size suggests that a park’s area is a poor factor in explaining visitation trends across socio-economic variables. These are detailed in Supplemental Table S6.

We also initially considered fitting other socio-economic independent variables into the same analysis. We did so in the hopes of gaining further insights on COVID-19’s impact towards park visitation. Some other independent variables that were considered included median income and median age. However, fitting them into same analysis resulted in high multi-collinearity. These are detailed in Supplemental Table S6. Multi-collinearity occurs when an independent variable is highly correlated with another independent variable in an analysis involving multiple independent variables^60^. This could consequently “undermine the statistical significance of an independent variable”^60^.

To mitigate concerns of multi-collinearity in our analysis involving different racial groups, we adopt the procedures outlined by Lewis-Beck and Lewis-Beck^60^. Lewis-Beck and Lewis-Beck recommends separating our analysis of each racial composition. This means that we would analyze the composition of non-whites, African-, Asian-, Hispanic-, and Native American with our other variables separately.

Finally, we considered analyzing the variables of income and age separately. However, the variables of income and age still resulted in high multi-collinearity amongst the existing independent variables. Furthermore, the different characteristics displayed amongst our analysis involving variables like income and age (compared to race) meant that our suggested random-effects gravity model is not a one-size-fits-all model for other analysis involving separate variables. These are detailed in Supplemental Table S6. For this reason, we hope to study variables like age and income in some of our future studies, using a different model.

## Supplementary Information

Supplementary Information.

## Data Availability

The SafeGraph Mobile Phone data are available for purchase at SafeGraph but commercial restrictions apply to the availability of these data, which were used under license for the current study, and so are not publicly available. Data are however available and could be downloaded for free for academics upon request and with permission from SafeGraph at https://www.safegraph.com/academics. The data for the geographical boundaries of all the national parks are openly available at National Parks Service Website at https://public-nps.opendata.arcgis.com/datasets/nps-boundary. The population and racial demographic data are available for purchase from the American Community Survey at https://www.socialexplorer.com/tables/ACS2019_5yr but commercial restrictions apply to the availability of these data, which were used under license for the current study, and so are not publicly available. Data are however could be made available and downloadable for academics upon registering for an account on Social Explorer at https://accounts.socialexplorer.com/signup using a valid institutional email. All data in this study are also included on in this published article’s Supplementary Information files (Supplementary Table S3).

## Code availability

Accession codes are available on GitHub at https://github.com/cja5553/national_park_visitation_analysis_w_mobile_phone_data.
