# Supplementary material for: COVID-19’s impact on visitation behavior to US national parks from communities of color: evidence from mobile phone data
Source: Sci Rep. 2022 Aug 4;12:13398. doi: 10.1038/s41598-022-16330-z (PMC9352905; doi:10.1038/s41598-022-16330-z)
Supplement: Supplementary file 1 — Supplementary Information. [file 41598_2022_16330_MOESM1_ESM.pdf]

Supplementary Information for: COVID-19's impact on visitation behavior to US national parks from communities of color - evidence from mobile phone data

S1 Explanation of percent change in visitation due to COVID-19

The percent change in visitation given $distance_{ij}$ travelled following the COVID-19 pandemic could be expressed as: $[\beta_1 + \beta_6 * \ln(distance)]\,\%$

S1.1 Derivation

This analysis concerns our use of interaction terms with variables on a logarithmic scale.

Because we are concerned with coefficients associated with $distance_{ij}$ and $COVID\_era$, we could deduce the equation to the following:

$$\ln(visitation_{ijt}) = \beta_0 + \beta_1 * (COVID\_era) + \beta_3 * \ln(distance_{ij}) + \beta_6 * [COVID\_era \times \ln(distanceij)]$$

Taking the derivative with respect to $COVID\_era$, we obtain:

$$\frac{1}{visitation_{ijt}} \frac{\partial visitation_{ijt}}{\partial COVID\_era} = \beta_1 + \beta_6 * [\ln(distance_{ijt})]$$

Multiplying by $COVID\_era$ we get,

$$\frac{COVID\_era}{visitation_{ijt}} \frac{\partial visitation_{ijt}}{\partial COVID\_era} = (COVID\_era) * \beta_1 + (COVID\_era) * \beta_6 [\ln(distance_{ijt})]$$

Since $COVID\_era = 1$ resulting from COVID-19, we obtain

$$\frac{COVID\_era}{visitation_{ijt}} \frac{\partial visitation_{ijt}}{\partial COVID\_era} = \beta_1 + \beta_6 [\ln(distance_{ijt})]$$

where the Left-hand-side is the elasticity of $visitation_{ijt}$ with respect to $COVID\_era$

Derivation partly adapted from Masterov.[1].

S2 Explanation of change in visitation for every percent change in race due to COVID-19

We would witness a $[\beta_5 - \beta_8 * \ln(distance_{ij})]\,\%$ change in visitation for every percent change of proportion of $race_i$ due to the COVID-19 pandemic.

S2.1 Derivation

This analysis concerns our use of interaction terms with variables on a logarithmic scale.

We have to consider all significant variables and interaction terms involving variable $race_{ijt}$. Thus, given

$$\ln(visitation_{ijt}) = \beta_0 + \beta_2[\ln(race_i)] + \beta_5[COVID\_era \times \ln(race_i)] + \beta_7[\ln(distance_{ij}) \times \ln(race_i)] + \beta_8[(COVID\_era \times \ln(distance_{ij}) \times \ln(race_i)]$$

Thus differentiating it with respect to $race_i$, we obtain:

$$\frac{\partial visitation_{ijt}}{\partial race_i} \frac{1}{visitation_{ijt}} = \frac{\beta_2}{race_i} + \frac{\beta_5(COVID\_era)}{race_i} + \frac{\beta_7 [\ln(distance_{ij})]}{race_i} + \frac{\beta_8(COVID\_era)[\ln(distance_{ij})]}{race_i}$$

Multiplying by $(race_i)$, we obtain:

$$\frac{\partial visitation_{ijt}}{\partial race_i}\frac{race_i}{visitation_{ijt}} = \beta_2 + \beta_5 * (COVID\_era) + \beta_7 * \ln(distance_{ij})$$
$$+\beta_8 * [(COVID\_era) * (\ln(distance_{ij})]$$

where the Left-hand-side is the elasticity of $visitation_{ijt}$ with respect to $COVID\_era$.

Because $COVID\_era = 1$ for after COVID-19 and $COVID\_era = 0$ for before COVID-19, we can calculate the difference of that before and after COVID-19 as:

$$[\beta_2 + \beta_5 + \beta_7 * \ln(distance_{ij}) + \beta_8 * \ln(distance_{ij})] - [\beta_2 + \beta_7 * \ln(distance_{ij})] = \beta_5 + \beta_8 * \ln(distance_{ij})$$

S3 Explanation of change in visitation for every percent increase in race

This analysis concerns our use of interaction terms with variables on a logarithmic scale.

The percent change in visitation for every percent increase in $race_i$ expressed as: $[\beta_2 + \beta_7 * \ln(distance)]\,\%$

S3.1 Derivation

Because we are concerned with significant terms associated with $race_i$, we could deduce the equation to the following:

$$\ln(visitation_{ijt}) = \beta_0 + \beta_2 * \ln(race_i) + \beta_7 * [\ln(race_i) \times \ln(distance_{ij})]$$

Taking the derivative with respect to $race_i$, we obtain:

$$\frac{1}{visitation_{ijt}}\frac{\partial visitation_{ijt}}{\partial race_i} = \frac{\beta_2}{race_i} + \frac{\beta_7 * \ln(distance_{ijt})}{race_i}$$

Multiplying by $race_i$ we get,

$$\frac{race_i}{visitation_{ijt}}\frac{\partial visitation_{ijt}}{\partial race_i} = \beta_2 + \beta_7 * \ln(distance_{ijt})$$

where the Left-hand-side is the elasticity of $visitation_{ijt}$ with respect to $race_i$

Derivation partly adapted from Masterov[1].

Figure S1. Comparison of annual visitation records before the COVID-19 (2019 March to 2020 Feb) and after COVID-19 (2020 March to 2021 Feb)

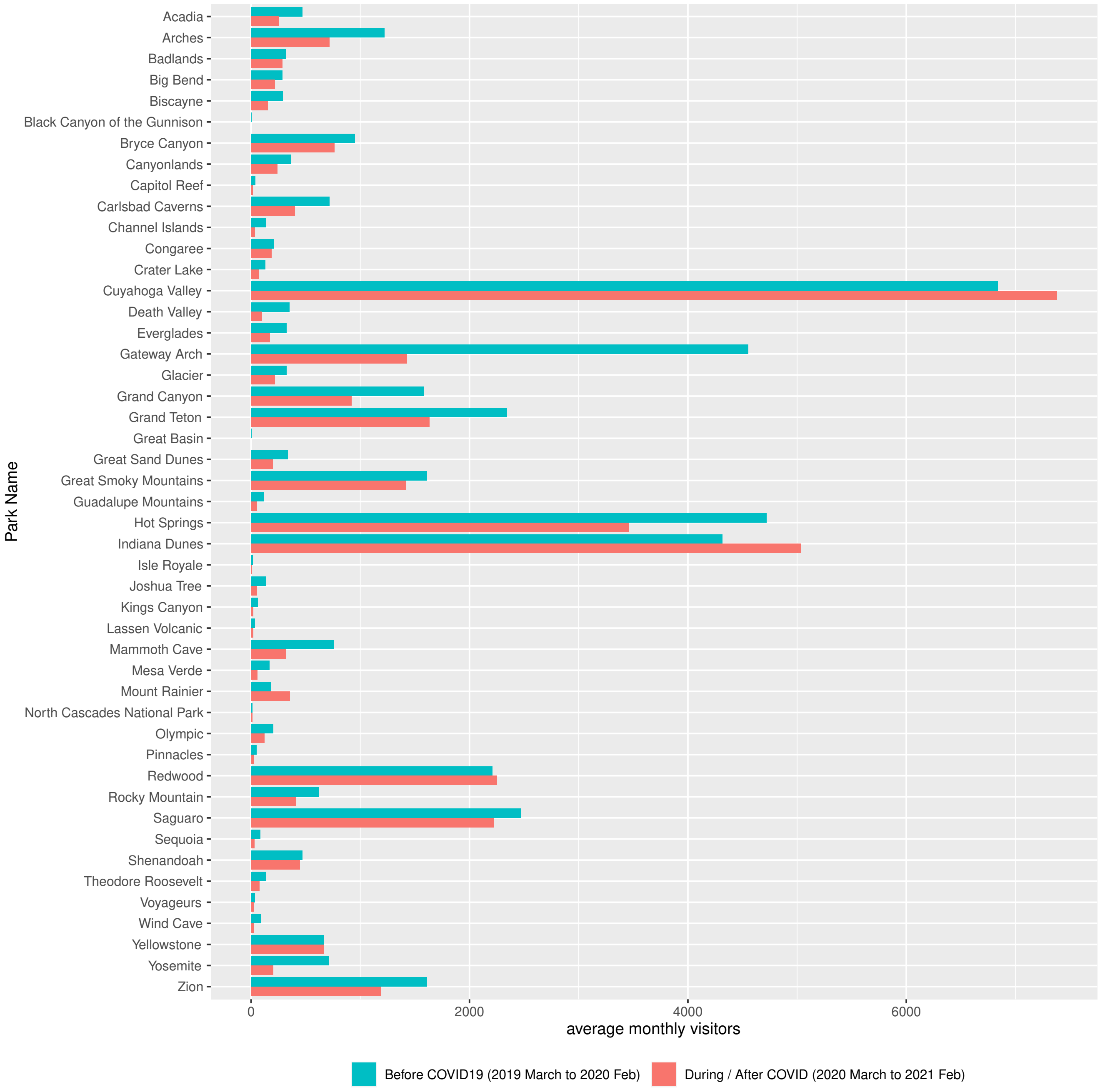

Figure S2. Raw estimates of the relationship between distance and COVID-era to complement Figure 2a of the manuscript. Figure 2a displays the interaction plot between distance and covid-era.

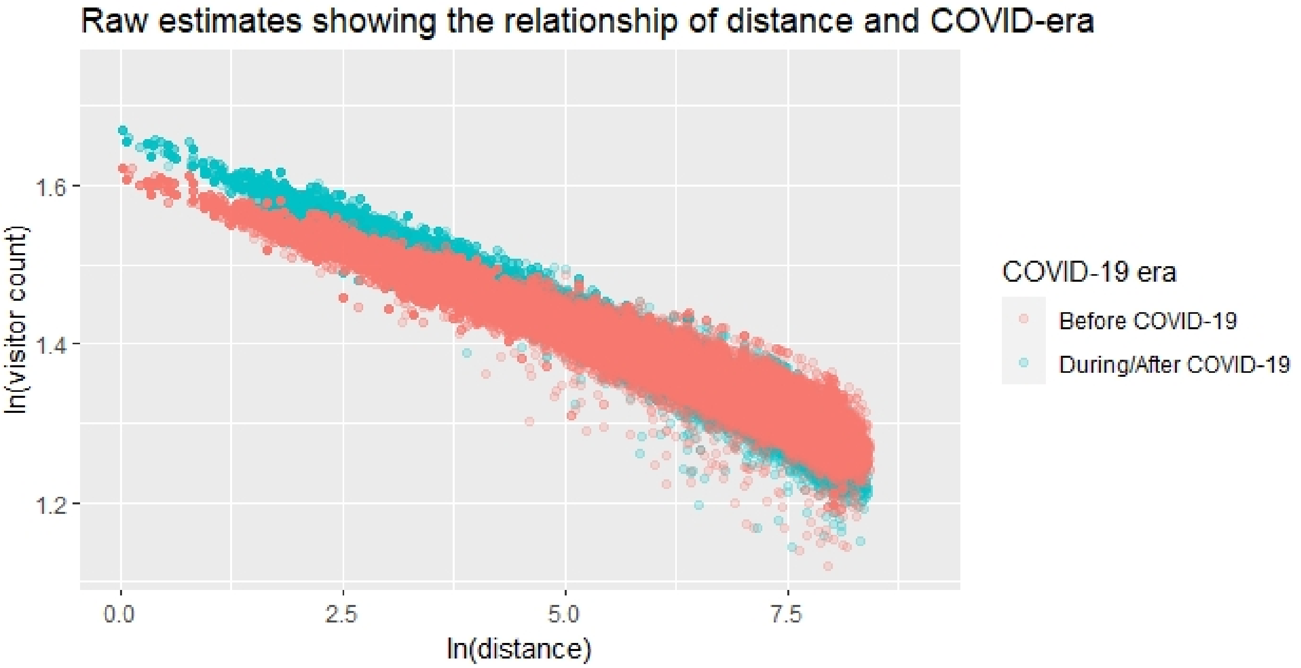

Table S1. Summary of the definition of variables

Variable Name	Variable type	Definition
Visitation_{ijt}	Dependent	The number of visitors from i^{th} census block to j^{th} national park during t^{th} month
COVID era	Independent	Any date before March 2021 is classified as "before COVID-19", whereas any date after March 2021 is classified as "After/During COVID-19". Therefore, FALSE=(before COVID-19) while TRUE=(During/After COVID). March 2021 is selected because it was the month whereby WHO declared COVID-19 a global pandemic[2], which was followed by travel restrictions due to lockdowns[3].
distance_{ijt}	Independent	The distance, in kilo-meters, traveled by visitors from their census block group (i) to the respective National Park (j). The distance was calculated through the Haversine formula[4] between the POIs of the Census Block Group's Centroid and that of the National Park's.
$\frac{\text{population}_i}{1000}$ (population per 1000)	Independent	The population of the census block group (i) divided by 1000.
race_i	Independent	The percent of each specified racial demographics situated in each respective census block group (i). These racial demographics include the proportion of non-whites, African-, Hispanic-, Asian-, and Native-Americans in each census block group.

Table S2. Descriptive Statistics

Variable	Mean	Min	Max	n
$\frac{population}{1000}$ (population per 1000)	1.783	0.002	59.947	86140 Census Block Groups
Proportion of Non-whites	0.2129	0	1	86140 Census Block Groups
Proportion of African-Americans	0.09221	0	1	86140 Census Block Groups
Proportion of Hispanics	0.13054	0	1	86140 Census Block Groups
Proportion of Asian-Americans	0.04965	0	1	86140 Census Block Groups
Proportion of Native-Americans	0.007474	0	1	86140 Census Block Groups
Distance	802.557	0.155	4533.039	221526 mobile-phone records
Visitation	4.933	4	621	221526 mobile-phone records

Table S3. Data Sources and Variables

Variable	Data Source	Time Range
Visitor Counts and Census Block Group Origins of Visitors	SafeGraph Patterns Dataset[5,a]	Jan 2018 to Apr 2021
Shape Files of each National Park	National Park Service Land Resources Division OpenData[6,b]	-
Racial Demographics of each visitors Census Block Group Origins	American Community Survey[7,c]	2015 to 2019

[a] Freely provided upon request from SafeGraph at https://www.safegraph.com/academics.
Note: SafeGraph provides the option of using "visitor_daytime_cbg" or "visitor_nighttime_cbg". We opted to use "visitor_daytime_cbg" because the computation of "visitor_nighttime_cbg" was changed on May 2020, which coincided very close to the start of COVID-19. So any changes detected from COVID-19 could have very well been confounded by changes resulting from SafeGraphs methodological changes[8].
[b] Available on the National Parks Service Website at https://public-nps.opendata.arcgis.com/datasets/nps-boundary
[c] Available at the American Community Survey website at https://www.socialexplorer.com/tables/ACS2019_5yr/R12847148

Table S4. comparison of statistical tests to determine the best model amongst pooled, fixed, and random effects model.

Test Name	Model comparisons (H_A)	Test Statistic					
		Race=(none)	Race = non-white	Race = African American	Race = Hispanic	Race = Asian American	Race = Native Americans
F-test[9]	Fixed vs Pooled (In favor of Fixed)	7.7171***	7.7386***	7.5969***	7.6965***	7.7729***	7.812***
Hausman Chi-square Test[10]	Fixed vs Random (In favor of Fixed)	8.8546	6.8134	1.9159	11.753	10.1	21.28*
(BP) Lagrange Multiplier Test[11]	Random vs Pooled (In favor of Random)	198***	190.78***	182.4***	188.68***	193.23***	198***

Signif. codes: 0 ‘***’ 0.001 ‘**’ 0.01 ‘*’ 0.05 ‘.’ 0.1 ‘ ’ 1

Table S5. Testing of the assumptions of log-linearity and multi-collinearity for our selected model

Assumption	Test Statistic	Model Tested						Comments
		race=(none)	race=non-white	race=african-american	race=hispanics	race=asian-american	race=native-american	
log-linearity	GAM plots between the logarithmic values of the dependent & independent variable[12]	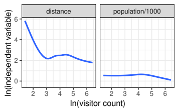	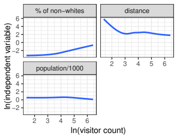	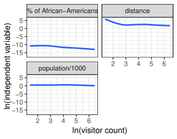	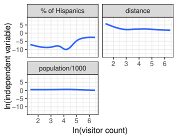	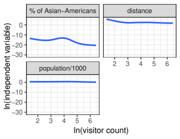	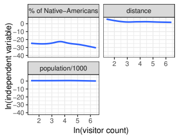	All the plots display a trend that is generally linear in nature, thereby passing the assumption of log-linearity[12].
multi-collinearity	$GVIF^{\frac{1}{(2*df)}}$ value range[13]	1.03 to 3.26	1.00 to 4.01	1.00 to 4.73	1.00 to 3.97	1.00 to 4.91	1.00 to 7.82	The $GVIF^{\frac{1}{(2*df)}}$ values fall within the acceptable range to pass the assumption of multicollinearity[13–15].

Table S6. A summary of the other variables and methods that we considered in our study.

variables of other models considered	Statistical Test Results	Concerns
Using the park’s area (in km^2) instead of distance travelled (in km) as part of the gravity model	Within R^2 ranging from 0.005 to 0.006<br>Between R^2 ranging 0.702 to 0.984	The R^2 values are substantially lower compared to using the distance travelled. This suggests a parks area is a poor factor in explaining park visitation trends across socio-economic variables.
Median income, COVID-19, and Distance	Highest GVIF^(1/(2*Df)) Score of 21.066459<br>F = 7.1122*** for F-test<br>chisq = 11.944 for Hausman Test<br>chisq = 152.12*** for LM test	While the F-[9], Hausman-[10], and LM[11]-Test validate the use of the random-effects gravity model, the high GVIF^(1/(2*Df)) scores suggest high multi-collinearity amongst the independent variables[15]
Median age, COVID-19, and Distance	Highest GVIF^(1/(2*Df)) score of 16.409974<br>F = 7.1122*** for F-test<br>chisq = 37.581*** for Hausman Test<br>chisq = 155.73*** for LM test	The F-[9], Hausman-[10], and LM[11]-Test scores suggest that the random-effects gravity model may not be suited for this analysis. While our GVIF^(1/(2*Df)) scores are lower than that of the median income analysis, it is still concerningly high, suggesting potential multi-collinearity[15].
Median income, race, COVID-19, and Distance	Highest GVIF^(1/(2*Df)) score of 33.441412<br>F = 7.2113*** for F-test<br>chisq = 210.12*** for Hausman Test<br>chisq = 157.98*** for LM test	The F-[9], Hausman-[10], and LM[11]-Test scores suggest that the random-effects gravity model may not be suited for this analysis. The GVIF^(1/(2*Df)) scores are concerningly high, suggesting potential multi-collinearity[15].
Median age, race, COVID-19, and Distance	Highest GVIF^(1/(2*Df)) score of 22.194502<br>F = 14.862*** for F-test<br>chisq = 37.581*** for Hausman Test<br>chisq = 155.73*** for LM test	The F-[9], Hausman-[10], and LM[11]-Test scores suggest that the random-effects gravity model may not be suited for this analysis. The GVIF^(1/(2*Df)) scores are concerningly high, suggesting potential multi-collinearity[15]

Signif. codes: 0 ‘***’ 0.001 ‘**’ 0.01 ‘*’ 0.05 ‘.’ 0.1 ‘ ’ 1

Codes and other resources used to analyze the data and run the models are available on Github (https://github.com/cja5553/national_park_visitation_analysis_w_mobile_phone_data)

References

1. Masterov, D. V. How to interpret interaction in log log models. Cross Validated (2019). URL:https://stats.stackexchange.com/q/405720 (version: 2019-04-30), https://stats.stackexchange.com/q/405720.
2. Cucinotta, D. & Vanelli, M. W.H.O declares covid-19 a pandemic. *Acta Bio Medica: Atenei Parmensis* **91**, 157 (2020).
3. Lamb, T. L. *et al.* A qualitative analysis of social and emotional perspectives of airline passengers during the covid-19 pandemic. *J. Air Transp. Manag.* **94**, 102079 (2021).
4. Robusto, C. C. The cosine-haversine formula. *The Am. Math. Mon.* **64**, 38–40 (1957).
5. SafeGraph. Mobile phone data from safegraph inc. (2021).
6. Division, N. P. S. L. R. National park service tract and boundary data (2021).
7. 2019 american community census survey data 5 year estimate (2019).
8. SafeGraph. Safegraph documentation (2021).
9. Torres-Reyna, O. Getting started in fixed/random effects models using r. *Data & Stat. Serv. Princet. Univ.* (2010).
10. Hausman, J. A. Specification tests in econometrics. *Econom. J. econometric society* 1251–1271 (1978).
11. Breusch, T. S. & Pagan, A. R. A simple test for heteroscedasticity and random coefficient variation. *Econom. J. econometric society* 1287–1294 (1979).
12. Kassambara, A. *Machine learning essentials: Practical guide in R* (Sthda, 2018).
13. Fox, J. & Monette, G. Generalized collinearity diagnostics. *J. Am. Stat. Assoc.* **87**, 178–183 (1992).
14. Özkale, M. R. The red indicator and corrected vifs in generalized linear models. *Commun. Stat. Comput.* **50**, 4144–4170 (2021).
15. Allen, M. P. The problem of multicollinearity. *Underst. regression analysis* 176–180 (1997).
